# Moth olfactory receptor neurons adjust their encoding efficiency to temporal statistics of pheromone fluctuations

**DOI:** 10.1371/journal.pcbi.1006586

**Published:** 2018-11-13

**Authors:** Marie Levakova, Lubomir Kostal, Christelle Monsempès, Vincent Jacob, Philippe Lucas

**Affiliations:** 1 Institute of Physiology of the Czech Academy of Sciences, Prague, Czech Republic; 2 Institute of Ecology and Environmental Sciences, INRA, Versailles, France; 3 Peuplements végétaux et bioagresseurs en milieu végétal, CIRAD, Université de la Réunion, Saint Pierre, Ile de la Réunion, France; University of Cambridge, UNITED KINGDOM

## Abstract

The efficient coding hypothesis predicts that sensory neurons adjust their coding resources to optimally represent the stimulus statistics of their environment. To test this prediction in the moth olfactory system, we have developed a stimulation protocol that mimics the natural temporal structure within a turbulent pheromone plume. We report that responses of antennal olfactory receptor neurons to pheromone encounters follow the temporal fluctuations in such a way that the most frequent stimulus timescales are encoded with maximum accuracy. We also observe that the average coding precision of the neurons adjusted to the stimulus-timescale statistics at a given distance from the pheromone source is higher than if the same encoding model is applied at a shorter, non-matching, distance. Finally, the coding accuracy profile and the stimulus-timescale distribution are related in the manner predicted by the information theory for the many-to-one convergence scenario of the moth peripheral sensory system.

## Author summary

Sensory neural systems of living organisms encode the representation of their environment with remarkable efficiency. We study the dynamic coding of naturalistic olfactory stimulation by pheromone-specific antennal neurons. The analysis reveals that the representation is optimal from several complementary information-theoretic perspectives. (1) Pheromone encounters are best detected if the concentration follows the naturally intermittent time course. (2) Antennal neurons dynamically adjust to the local stimulus statistics. (3) The coding accuracy profile and the stimulus-timescale distribution are in the relationship predicted by both information theory and statistical estimation theory.

## Introduction

**Fig 1:**
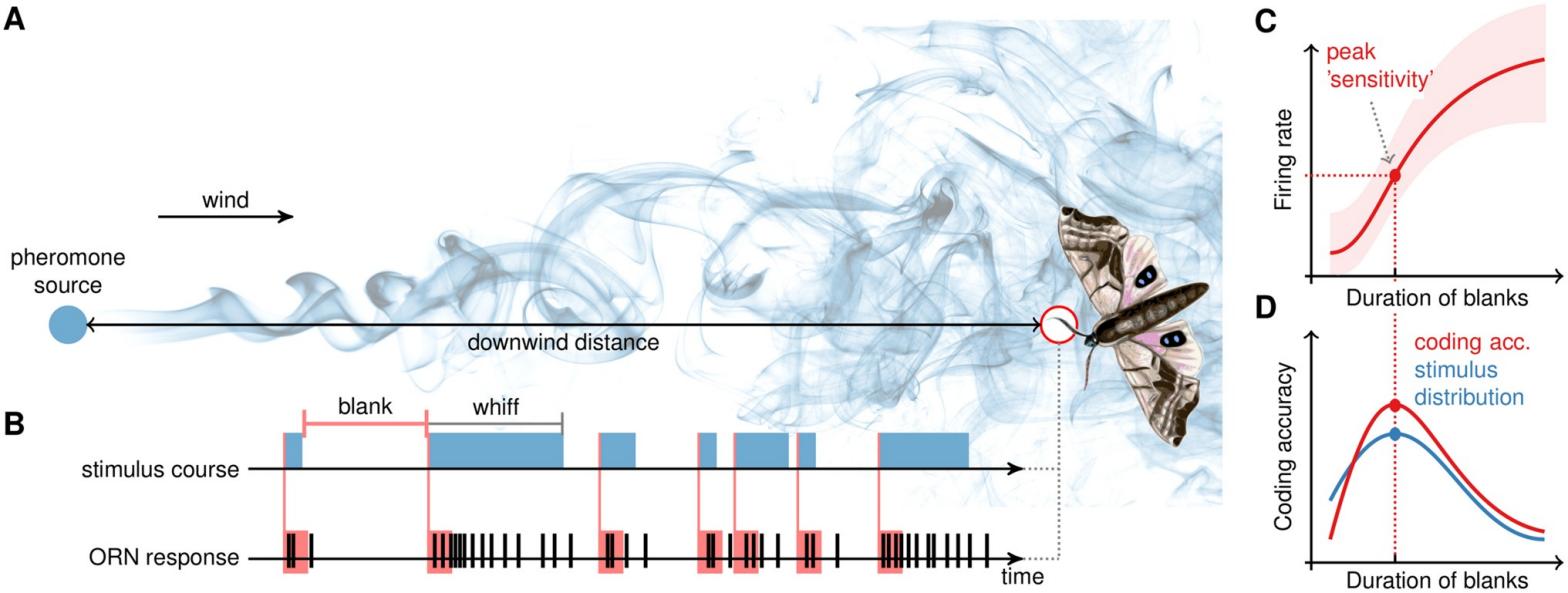
Graphical abstract. (**A**) Atmospheric turbulence governs the complicated non-homogeneous dispersion of a pheromone, which is detected by specialized olfactory receptor neurons (ORNs) located on the moth antennae (red circle). (**B**) A typical time course of the pheromone stimulation at a given distance from the source is intermittent. The signal consists of *blanks*, intervals of zero local concentration due to the passage of clean-air pockets, and of *whiffs*, intervals of pheromone presence. The statistics of blanks and whiffs describes the spatio-temporal structure of the turbulent plume. (**C**) A simple encoding model of a whiff encounter is given by the dependence of the firing rate (measured within a period after the whiff onset) on the preceding blank duration, the *duration-rate relationship*. The coding sensitivity of the whiff encounter is determined from the slope of the mean response and the response variability. In order to detect the pheromone optimally, the efficient coding hypothesis predicts the ORN to adjust its encoding sensitivity to the local stimulus conditions by adjusting the duration-rate relationship. (**D**) We observe that encoding properties of ORNs are adjusted to match the local distribution of blank durations. Particularly, *i)* the maximal sensitivity corresponds to the most frequent blank duration (*stimulus timescale*), *cf*. Figs 4 and 5; *ii)* the average decoding accuracy is largest for the matching stimulus-timescale distribution (Fig 6); and *iii)* the profile of the coding accuracy matches the stimulus-timescale distribution optimally from an information-theoretic point of view (Figs 7 and 8). (The figure is meant only as an illustration of the studied problem and does not represent the measured data.)

Orienting towards food and mates in insects is an olfactory-controlled behavior that relies on detecting odorant molecules delivered from the source. Atmospheric turbulence causes strong mixing of air and creates a wide spectrum of spatio-temporal variations in the signal (Fig 1). The largest eddies may be hundreds of meters in extent and take minutes to pass a fixed point, while the smallest spatial variations could have a size of less than a millimeter and last for milliseconds [1–3]. The mean concentration of the odorant decreases with distance from the source, however, a signal with a large instantaneous magnitude can be found in a wide range of distances from the source, though their frequency decreases with distance [1]. Hence, an important characteristic of the detected signal is its intermittency, i.e., the fraction of time during which the odorant can be detected [1, 4, 5]. Rapid behavioral responses of male moths tracking plumes in turbulent flows [6] and the ability of neurons from the first two layers of the olfactory system to encode the temporal dynamics of pheromone plumes at any distance from the source [7] suggest efficient coding of olfactory plume dynamics.

**Fig 2:**
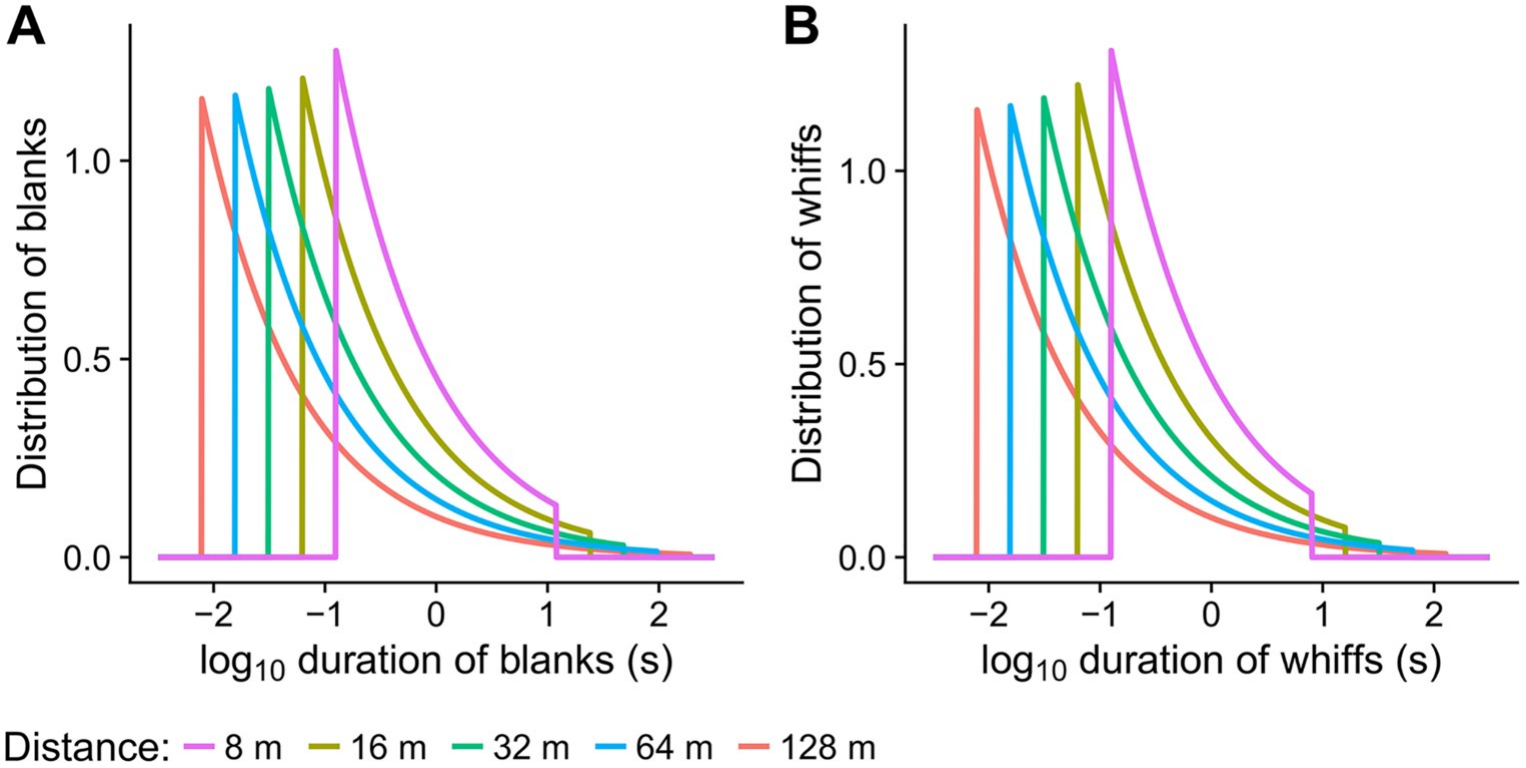
The temporal structure of the pheromone plume at a given downwind distance from the source (color) is characterized by the distribution of blanks and whiffs, which are independent. (**A**) Distribution of *blanks*, intervals without pheromone detection. (**B**) Distribution of *whiffs*, intervals with detectable pheromone presence.

Recently, the statistical distributions of odorant fluctuations was described [3], namely the statistics of time intervals with the presence of an odorant at a given point in space, denoted as *whiffs*, and intervals when the odorant concentration is zero, *blanks*. The distributions of whiff and blank durations change with the distance of a detector from a source (Fig 2) and provide together an important statistical description of the local spatio-temporal properties of the pheromone plume.

The local statistics of many natural stimuli differs from the average global distribution, and the limited coding range of neurons does not cover the wide range of all possible stimulus values [8–10]. The efficient coding hypothesis [11] states that neuronal responses are adjusted, through evolutionary and adaptive processes, to optimally encode such stimulus statistics that match the local sensory environment [12–15]. The hypothesis thus predicts that coding accuracy is highest for the most commonly occurring events to minimize overall decoding error. Such situations have been reported in auditory coding of sound intensity [8, 9, 16, 17], of interaural level differences [18] and time differences [19], and also for primary visual cortex [10] and primary somatosensory cortex [20]. To the best of our knowledge, an analogous study has not been done yet in odor detection, partially due to the difficulties associated with the description of the natural stimulus statistics and its changes [2–4, 21].

In this work, we study how pheromone-sensitive olfactory receptor neurons (ORNs) adjust their responses to the local stimulus statistics (Fig 1). Our results show that ORN responses are adjusted in such a way that pheromone encounters are encoded best after blanks that have the most common duration. We also found that the average accuracy of pheromone detection is better if an encoding scheme is adapted to the stimulus statistics of a particular distance from the source than if the same scheme is applied closer to the source. In addition, ORNs’ coding properties support an idea of efficient population information transmission from the ORNs to the antennal lobe neurons.

## Materials and methods

### Insects

Experiments were performed with laboratory-reared adult males of *Agrotis ipsilon* fed an artificial diet [22]. Pupae were sexed, and males and females were kept separately at 22 °C in an inversed light-dark cycle (16 h–8 h light-dark photoperiod). Adults were given access to 20% sucrose solution *ad libitum*. Experiments were performed on virgin 4- or 5-day-old (sexually mature) males.

### Electrophysiology (single-sensillum recordings)

Insects were restrained in a Styrofoam block with the head protruding. One antenna was fixed with adhesive tape on a small support. Electrodes were made from electrolytically sharpened tungsten wires (TW5-6, Science Products, Hofheim, Germany). The recording electrode was inserted at the base of a long pheromone-responding sensillum trichodeum located on an antennal branch. The reference electrode was inserted in the antennal stem. The electrical signal was amplified (×1000) and band-pass filtered (10 Hz–5 kHz) with an ELC-03X (npi electronic, Tamm, Germany), and sampled at 10 kHz via a 16-bit acquisition board (NI-9215, National Inst., Nanterre, France) under Labview (National Inst.). One sensillum was recorded per insect.

### Virtual olfactory environment: Spatio-temporal character of the pheromone plume and stimulus distribution

ORNs were stimulated with the major sex pheromone component of *A. ipsilon*, (Z)-7-dodecenyl acetate (Z7-12:Ac). Pheromone stimuli were diluted in decadic steps in hexane and applied on a filter paper introduced in a Pasteur pipette at doses ranging from 10^−6^ to 10^0^ ng. The antenna was constantly superfused by a humidified and charcoal-filtered air stream (70 L ⋅ h^−1^). Air puffs (10 L ⋅ h^−1^) were delivered through a calibrated capillary (Ref. 11762313, Fisher Scientific, France) positioned at 1 mm from the antenna and containing the odor-loaded filter paper (10 × 2 mm). An electrovalve (LHDA-1233215-H, Lee Company, France) was controlled by custom Labview programs reading sequences generated using Matlab scripts. The time resolution of the sequences was 1 ms. The characteristic response time of the valves, i.e. the time to go from open to close (close to open) is <5 ms.

Durations of whiffs (puffs) and blanks were set to mimic the turbulent dynamics of the odorant plume in a real environment according to the model by Celani et al. [3] at 5 virtual downwind distances from the pheromone source (*d* = 8, 16, 32, 64 and 128 m). The virtual crosswind distance was always 0, hence the positions were virtually in the centre of the pheromone plume. The geometric progression of distances was chosen to emphasize the effect of turbulence on puff/non-puff statistics. Other parameters of the model were *U* = 1 m ⋅ s^−1^ (mean wind velocity), *δU* = 0.1 m ⋅ s^−1^ (wind fluctuations), *a* = 0.1 m (size of the pheromone source), *χ* = 0.4 (intermittency factor), yielding the probability density function of blank (B) and whiff (W) durations

$$\begin{array}{c} f_{B}\left(x\right)=\frac{x^{-3/2}}{2(1/\sqrt{\tau }-1/\sqrt{T_{B}})},\;x\in \left[\tau ,T_{B}\right], \end{array}$$ (1)

$$\begin{array}{c} f_{W}\left(x\right)=\frac{x^{-3/2}}{2(1/\sqrt{\tau }-1/\sqrt{T_{W}})},\;x\in \left[\tau ,T_{W}\right], \end{array}$$ (2)

where *τ* = *a*^2^*d*/[*d*(*δU*)^2^] is the shortest possible blank (whiff), *T*_*W*_ = *d*/*U* is the longest possible whiff and *T*_*B*_ = *T*_*W*_(1/*χ* − 1) is the longest possible blank. Throughout the paper, we report the results with respect to decadic logarithms of the durations of blanks. The logarithm of a blank represents a transformed random variable *Y* = *g*(*B*) = log_10_ *B* and hence the corresponding probability density function is derived using the formula

$$\begin{array}{c} f_{Y}\left(y\right)=f_{B}(g^{-1}\left(y\right))|\frac{d}{dy}g^{-1}\left(y\right)|. \end{array}$$ (3)

Plugging *g*^−1^(*y*) = 10^*y*^ and d*g*^−1^/*dy* = 10^*y*^ ln 10 yields

$$\begin{array}{c} f_{\log_{10}B}\left(x\right)=\frac{10^{-\frac{x}{2}}\ln10}{2(1/\sqrt{\tau }-1/\sqrt{T_{B}})},\;x\in \left[\log_{10}\tau ,\log_{10}T_{B}\right]. \end{array}$$ (4)

Sequences of whiffs and blanks were tested only once on a single recorded ORN. The dose of pheromone was constant throughout one recording session.

For the two largest virtual downwind distances from the source, 64 and 128 m, we selected the generated sequences, excluding those exhibiting extremely long stimuli, which led to the complete shutdown of ORN spiking activity. Thus, the statistics for 64 and 128 m were biased from the pure turbulence by removal of extremely rare events (puffs >30 s).

### Data analysis

The data were analyzed using the R programming environment [23]. In total, we analyzed recordings of 217 moth ORNs obtained at 5 virtual distances and for 7 levels of pheromone dose. For each combination of virtual distance and pheromone dose we had 3-11 recordings of distinct ORNs, with the exception of 128 m and 1 ng dose, a category that was not studied because the occurrence of extremely long whiffs induced a complete interruption of the spiking activity at this high pheromone dose. Because the activity of ORNs is independent of other neurons [24], all the recordings obtained with a particular dose of pheromone and at a particular virtual distance were pooled and analyzed together.

#### Latency correction

The experimental setup induces latency between the valve opening and the actual pheromone delivery, mainly due to the time needed for the air to pass through the capilary to the sensillum. The response latency *θ* was estimated for each group of neurons stimulated under the same conditions (virtual distance and pheromone dose) using a nonparametric method [25, 26]. The estimate $\hat{\theta }$ was determined as

$$\begin{array}{c} \hat{\theta }=\max\left\{t\in \left[0,\tilde{t}\right]:{\hat{F}}_{T}\left(t\right)-{\hat{F}}_{W}\left(t\right)\leq 0\right\}, \end{array}$$ (5)

where ${\hat{F}}_{W}\left(t\right)$ is the empirical cumulative distribution function of the intervals between the last spike during the blank and the whiff onset (*w*_1_, *w*_2_, …, *w*_*n*_), ${\hat{F}}_{T}\left(t\right)$ is the empirical cumulative distribution function of the intervals between the whiff onset and the first subsequent spike (*t*_1_, *t*_2_, …, *t*_*n*_) and

$$\begin{array}{c} \tilde{t}=\arg\max_{t\in [0,t^{\left(n\right)}]}({\hat{F}}_{T}\left(t\right)-{\hat{F}}_{W}\left(t\right)), \end{array}$$ (6)

$$\begin{array}{c} t^{\left(n\right)}=\max\{t_{1},\ldots ,t_{n}\}. \end{array}$$ (7)

The mean latency was 26.1 ms, standard deviation 10.3 ms; latencies were decreasing with respect to the pheromone dose.

#### Decoding accuracy (Fisher information)

The response to each stimulation was determined from the number of spikes in a 150 ms time window starting from a stimulus onset, corrected for latency. This duration was chosen because it corresponds to the delay of behavioral responses of moths to pheromone stimuli [27]. Shorter and longer time windows were also applied, but with minor impact on the results. The relationship between the response and the duration of the blank immediately preceding the stimulus onset constitutes a *duration-response (duration-rate) function*. Since the duration-rate relationship was not stable towards the beginning and the end of the recording [7] (e.g. due to technical reasons and due to adaptive processes), we analyzed only responses observed between 100 and 500 s of each recording, which represent the activity in the adjusted state.

The Fisher information *F*(*s*) was used in a standard way to evaluate the decoding accuracy [28, 29]. Fisher information is defined as

$$\begin{array}{c} F\left(s\right)=\sum_{r}P\left(r|s\right){\left(\frac{d\;\text{ln}[P(r|s)]}{\text{d}s}\right)}^{2}, \end{array}$$ (8)

where *P*(*r*|*s*) is the probability of *r* spikes in the observation window given that the logarithm of the duration of the preceding blank is equal to *s*. The reciprocal of the Fisher information is the lower bound on the mean square error achieved when estimating *s* from the response *r*, hence the value of the Fisher information indicates the decoding accuracy. We used an approximation of the exact Fisher information commonly used in this context [8–10, 16, 20, 30]. The approximation has the form

$$\begin{array}{c} F\left(s\right)\approx \frac{\gamma^{\prime }{\left(s\right)}^{2}}{\sigma^{2}\left(s\right)}, \end{array}$$ (9)

where *γ*′(*s*) is the derivative of the duration-response function, and *σ*^2^(*s*) is the variance of the responses for given *s*. The duration-response function *γ*(*s*) was obtained from a cubic smoothing spline, i.e. the fitted curve is a piecewise polynomial of the third order minimizing the penalized sum of squares $\sum_{i=1}^{n}{\left[r_{i}-\gamma \left(s_{i}\right)\right]}^{2}+\lambda \int \gamma^{\prime \prime }{\left(s\right)}^{2}\;\text{d}s$. The applied values of λ yielded the trace of the smoother matrix (approximately equivalent to degrees of freedom) around 3. To determine the variance, the responses were ordered according to the length of the corresponding blank and divided into overlapping segments, each containing 10 responses. A robust estimator of the variance (*IQR*/1.349)^2^, where *IQR* is the inter-quartile range, was applied. The estimated variances were then smoothed by performing a local linear regression of order 1, so that a regression line was fitted locally to the estimated variances weighted by a tricubic function centered at each *s* and spanning 90% of the data range.

#### Average Fisher information

The average Fisher information, 〈*F*〉, gives the average decoding accuracy implied by the Fisher information *F*(*s*) across the stimulus statistics characterized by the distribution *f*_log_10_*B*_(*s*) and is defined as

$$\begin{array}{c} \left\langle F\right\rangle =\int F\left(s\right)f_{\log_{10}B}\left(s\right)\;\text{d}s. \end{array}$$ (10)

The Fisher information was averaged using the blank distribution of the matching virtual distance or a shorter non-matching distance. Virtual distances longer than the matching one could not be applied since the Fisher information is not defined for the whole range of possible blank durations.

#### Optimal stimulus distribution

The Jeffreys prior *p*_*J*_(*s*) is a timescale distribution defined as [31]

$$\begin{array}{c} p_{J}\left(s\right)\propto \sqrt{F(s)}. \end{array}$$ (11)

We determined the Jeffreys prior *p*_*J*_(*s*) for *s* in the interval *I* = [log_10_ *τ*, *α*(0.5)], where *α*(0.5) is the median value of the logarithm of the durations of blanks. The Jeffreys prior for *s* ≤ *α*(0.5) is then

$$\begin{array}{c} p_{J}\left(s\right)=\frac{1}{c}\sqrt{F(s)},\;c=2\int_{\log_{10}\tau }^{\alpha (0.5)}\sqrt{F(s)}\;\text{d}s. \end{array}$$ (12)

## Results

The experimental setup emulated the fluctuating delivery of pheromone at 5 virtual distances (8, 16, 32, 64, 128 m) from the pheromone source. Pheromone dose was set to one of 7 levels, (10^−6^ to 10^0^ ng) and a pheromone of constant concentration was released in puffs (whiffs), separated by blanks (see Materials and methods). The lengths of whiffs and blanks were generated randomly from the distributions of blanks and whiffs in real plumes [3] to mimic the natural pheromone fluctuations at a given downwind distance from the pheromone source. Henceforward, the distribution of blank durations is also referred to as the *stimulus-timescale distribution*. The durations of blanks and whiffs are restricted to the intervals [*τ*, *T*_*B*_] and [*τ*, *T*_*W*_], respectively (Eq 1). As the distance from the source increases, the range of possible blank (whiff) durations becomes wider (Fig 2), but the shortest blanks and whiffs always appear with the highest frequency.

### Encoding model: ORN stimulus-response relationship

**Fig 3:**
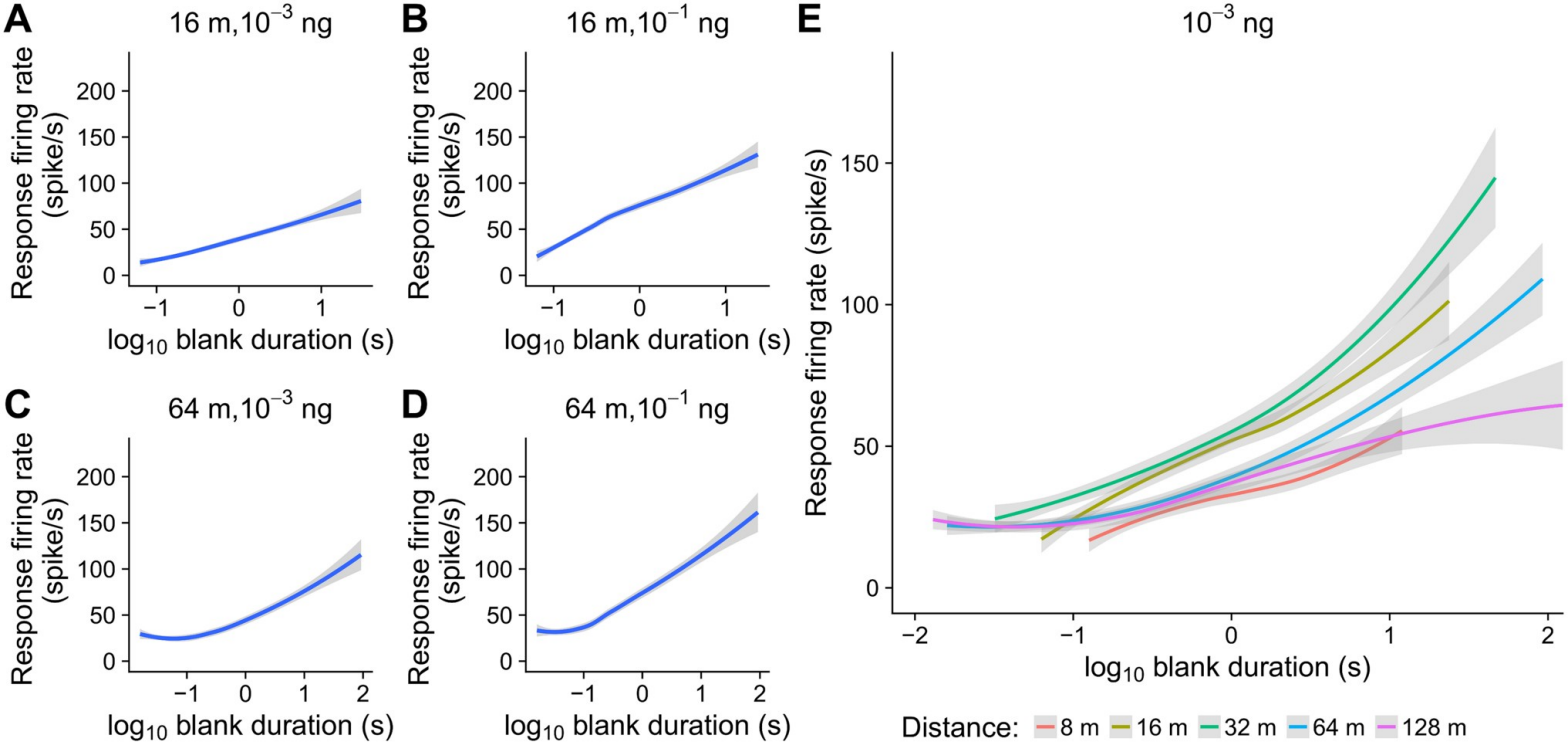
Responses of ORNs to pheromone encounter in dependence on the preceding blank duration (duration-rate relationships). The response is the average firing rate in a 150 ms time window starting with the whiff onset. (**A**, **B**) Responses to two pheromone doses (10^−3^ ng and 10^−1^ ng) at 16 m downwind distance from the pheromone source. Solid blue line represents the average, gray area indicates 95% confidence interval around the average. (**C**, **D**) Duration-rate relationships at 64 m downwind distance, the pheromone doses are same as in (A, B). Responses after longer blanks are more variable than responses preceded by shorter blanks. (**E**) Duration-rate relationship for all virtual distances with the pheromone dose 10^−3^ ng. The firing rate and the slope of duration-rate curves change systematically with the virtual distance, the variance is not affected much by the virtual distance.

ORN firing rate in response to a plume encounter was determined from the number of action potentials fired within the first 150 ms after each whiff arrival. The whiff onset is marked by a higher firing rate, which increases with the length of the immediately preceding blank (*duration-rate relationship*). The duration-rate relationship captures the sensitivity of the response with respect to the blank preceding the whiff onset and it is used as the encoding model for the whiff detection (Fig 3).

The duration-rate relationship was not stable throughout the whole recording. At the beginning, before the neurons became adjusted to the stimulation protocol, the responses were higher and became stabilized approximately after 100 s. Throughout the paper, we analyze only the behavior of ORNs in the adjusted state based on the recordings done between 100 s and 500 s.

The duration-rate relationship also changes with concentration of the odorant and the virtual distance. A higher pheromone dose leads to a higher maximum firing rate and higher slope of the duration-rate curve (Fig 3A–3D). The dependency on the virtual distance is less straightforward, nevertheless, we observe a systematic change of the slope of the curve, the maximum firing rate changes too, but the variance does not seem to be substantially affected (Fig 3E).

### Peak decoding accuracy is adjusted to the most frequent duration of blanks

We investigated what the ORN duration-rate relationship reveals about the coding accuracy of pheromone encounters. Decoding accuracy is commonly evaluated by means of the stimulus-reconstruction paradigm, that is, by answering how well an ideal observer may determine the stimulus value from a noisy neuronal response [32]. Coding accuracy is quantified and interpreted by employing Fisher information (see Materials and methods, Eq 8) in a standard way, i.e, we use the fact that the inverse of the Fisher information is the mean square error of decoding by an ideal observer [16, 28, 29, 33–38]. Hence, the value of the Fisher information reflects the ultimate decoding accuracy and the maximum of the Fisher information corresponds to the optimum conditions for decoding. The approximation of the Fisher information is the square of the slope of the mean response divided by the variance of responses at each point. Thus, Fisher information is high when the firing rate has a low variability and changes rapidly with respect to the blank duration.

**Fig 4:**
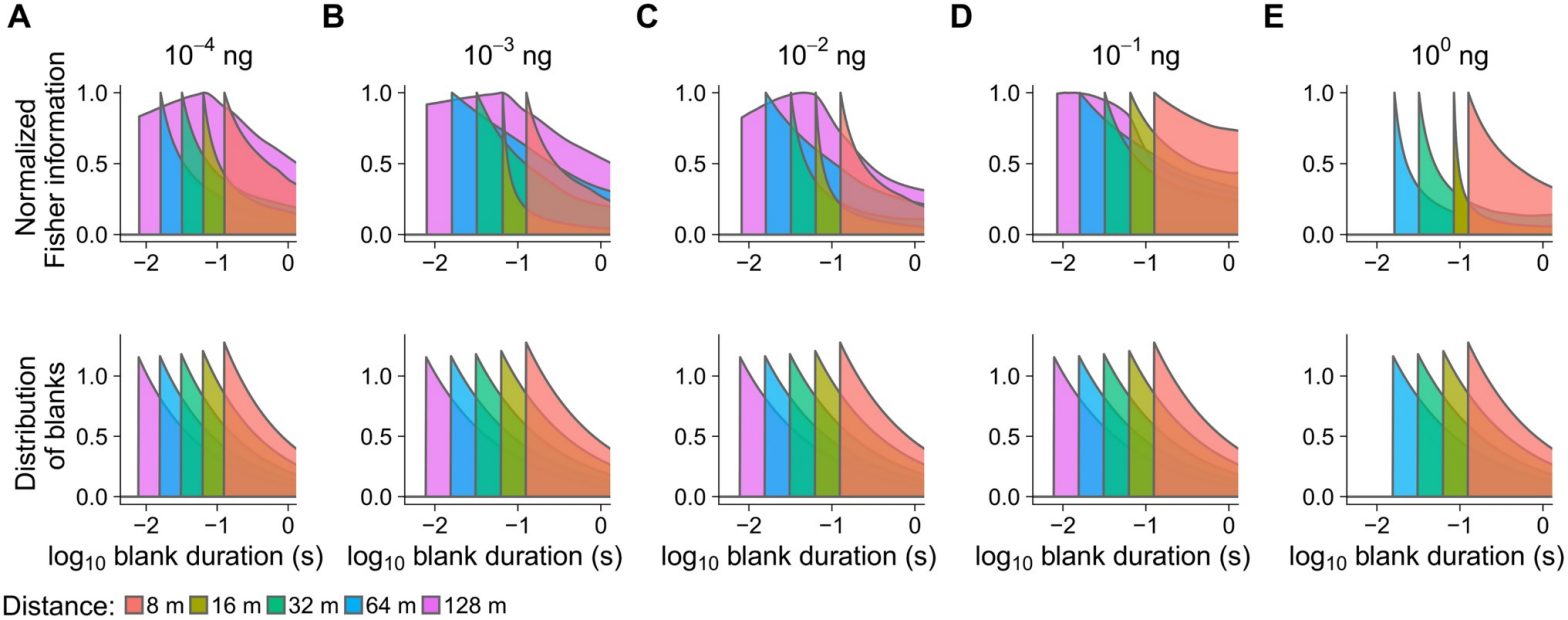
ORN coding precision of pheromone encounters is adjusted to the statistics of blanks in the plume. (**Top row**) Profiles of the coding accuracy (Fisher information) as a function of blank duration. The situation is shown for different pheromone doses (A–E) and virtual downwind distances from the source (color). Each Fisher information curve was individually scaled (normalized) to achieve that its maximum value is equal to 1. Stimulation by 10^0^ ng pheromone dose was not performed for 128 m. (**Bottom row**) The distributions of blanks for the corresponding distances. With the exception of the largest distance (128 m), the Fisher information profiles follow the distribution profiles, which means that the coding resources are distributed in agreement with the frequency of various blank durations. In particular, the maximal coding accuracy, indicated by the location of the maximum Fisher information, tends to occur at the mode of the corresponding distribution, *cf*. Fig 5. The adjustment results in an average coding accuracy optimized for the particular distance (Fig 6).

**Fig 5:**
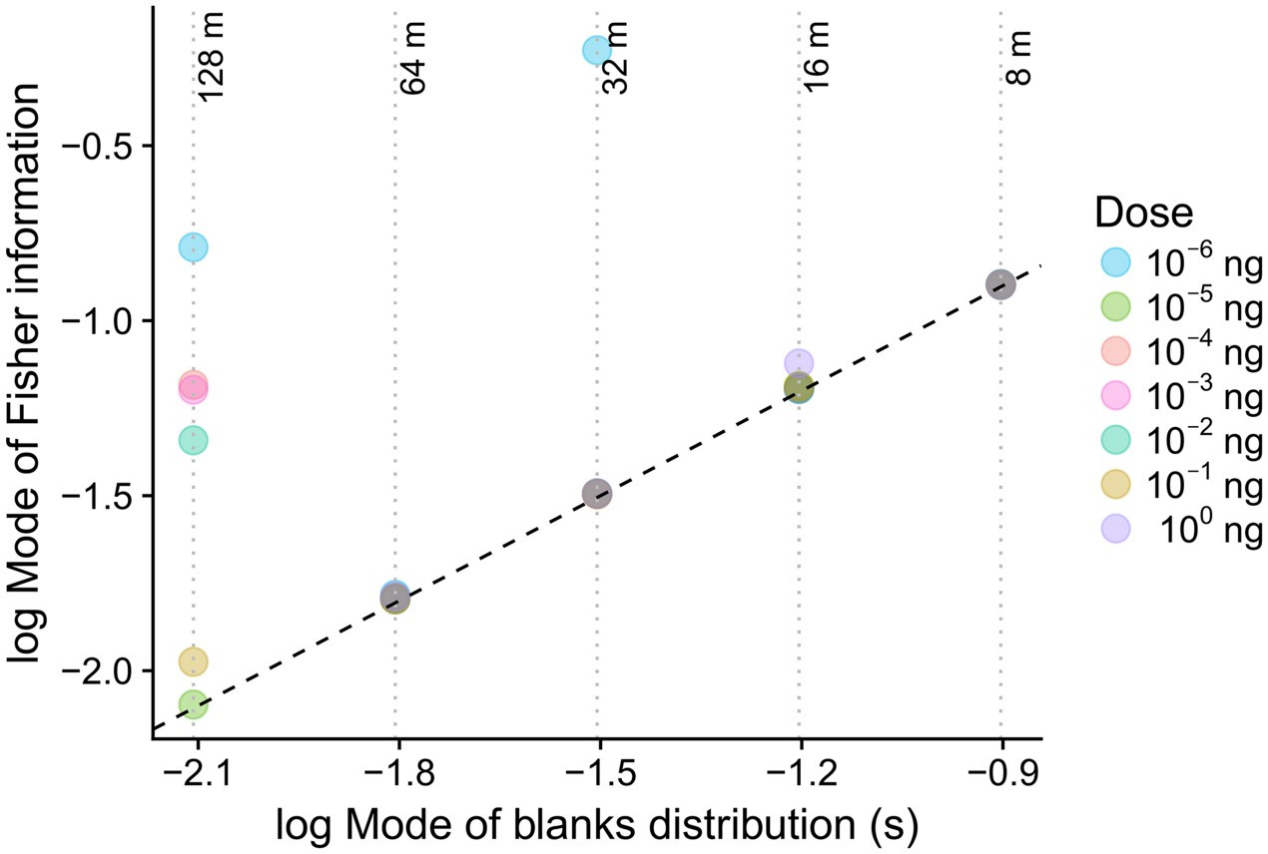
Positions of peaks in the ORN coding accuracy (mode of the Fisher information) tend to align with the most frequent duration of a blank. The exact matching (dashed line) occurs for almost all measured cases with the exception of the largest distance (128 m) and the lowest pheromone dose (10^−6^ ng).

We observe that the profiles of the coding accuracy (Fisher information) and of the stimulus-timescale distributions are matched (Fig 4) in the sense that the Fisher information reaches high values for events of stimulation with high probability of occurrence and has low values for rare stimulations. Most importantly, the modes of the corresponding Fisher informations and blank distributions coincide in most cases (Fig 5). The correlation between the mode of the Fisher information and the mode of the corresponding stimulus probability density function is *R* = 0.6. If the results obtained with the smallest dose of 10^−6^ ng are excluded, the correlation coefficient increases to *R* = 0.8. This implies that the sensitivity of neuronal responses is adjusted to the most frequent temporal patterns of stimulation.

### ORNs encode the temporal patterns at the given distance optimally

To assess the match of the complete Fisher information profiles to the stimulus-timescale statistics, we introduce the notion of *average decoding accuracy*. Each duration-rate relationship defines a specific encoding model for pheromone detection. We calculate the average decoding accuracy of an encoding model with respect to a given timescale distribution by integrating the whole profile of the Fisher information, where each value of the Fisher information is weighted proportionally to the frequency of the corresponding blank in the given timescale distribution (Eq 10 in Materials and methods).

**Fig 6:**
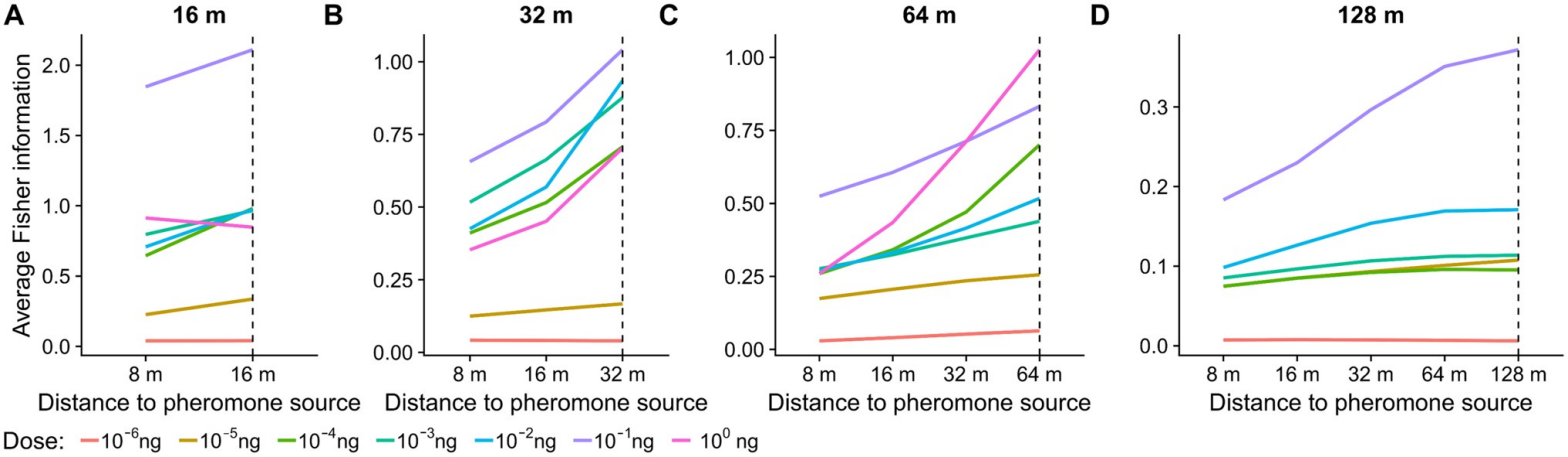
Overall coding accuracy (average Fisher information) is higher for the stimulus statistics of the matching distance than for mismatched statistics corresponding to shorter distances from the pheromone source. (**A**) The ORNs exposed to the temporal statistics of pheromone plume at the distance of 16 m achieve different coding accuracy in dependence on the pheromone dose (color). The average coding accuracy when the encoding model for 16 m is applied to the correct stimulus-timescale statistics of 16 m (dashed line) is greater than the average coding accuracy of the same encoding model when assuming a mismatched stimulus-timescale statistics at 8 m, for all pheromone doses except 1 ng. (**B–D**) Analogous results for ORNs adjusted to statistics of other distances (dashed). The coding performance is always best for the matching distance. Virtual distances longer than the matching one could not be applied since the Fisher information is not defined for the whole range of possible blank durations.

For each tested dose and virtual distance, we calculated the average decoding accuracy assuming *a)* the stimulus-timescale statistics to which the encoding model is adjusted and *b)* other stimulus-timescale statistics corresponding to nonmatching virtual distances to which the encoding model was not adjusted (Fig 6). Among all possible nonmatching distances, we considered only the distances shorter than the matching one, since the ranges of possible blanks at longer distances than the matching one are wider than the range of blanks for which the Fisher information was calculated, and therefore the average Fisher information cannot be determined.

We observe that the average decoding performance is highest for the stimulus-timescale statistics of the matching distance. And conversely, the same encoding model applied to the statistics of a non-matching distance always resulted in a lower overall decoding accuracy. The only exception was the encoding model obtained for 16 m with the largest dose of 1 ng.

### The profile of the Fisher information suggests optimal population coding

The first two layers of the moth olfactory system are organized so that the first-layer neurons (ORNs) converge onto a much smaller number of second layer neurons [24]. The signal-to-noise ratio (S/N) of the pooled signal increases with the square root of the number of pooled ORNs [39, 40]. Typically, hundreds of ORNs converge onto a single second-order neuron, resulting in a high S/N information transmission scheme. Assuming a homogeneous population of ORNs, information theory predicts that the optimal encoding scheme, i.e. a scheme that maximizes the mutual information between stimuli and responses [41–45], is such that the stimulus becomes a *Jeffreys prior* [46–51]. A Jeffreys prior is defined as a distribution that is proportional to the square root of the Fisher information. Vice versa, the Fisher information is then proportional to the second power of the stimulus distribution. Although the definition of the Jeffreys prior might evoke the idea that the stimulus distribution is to be adjusted in order to correctly correspond to the Fisher information, it is not the stimulus distribution, but the encoding model that must be tuned in order to establish this relation.

**Fig 7:**
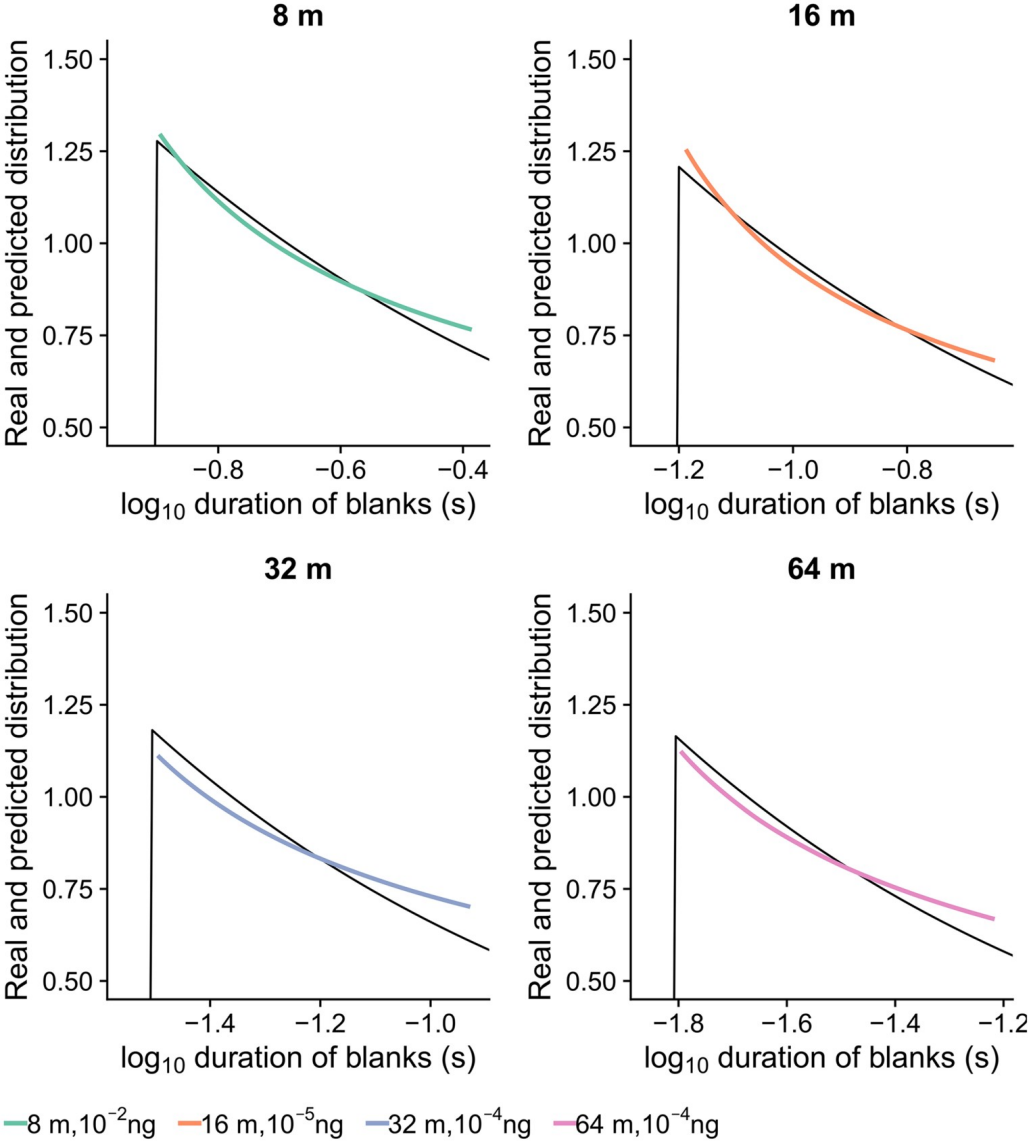
The distribution of blanks predicted by the information theory for optimal encoding in high S/N scenario (color) is close to the real distribution in the natural environment (black). The natural blanks distribution is very close to the Jeffreys prior (a distribution proportional to the square root of the Fisher information), suggesting that ORNs encode a whiff encounter optimally (transmit maximum information possible) if the simultaneous output of multiple independent ORNs is used for decoding. We speculate that such a setup is viable and in fact even corresponds to the basic anatomy of the moth peripheral olfactory system.

**Fig 8:**
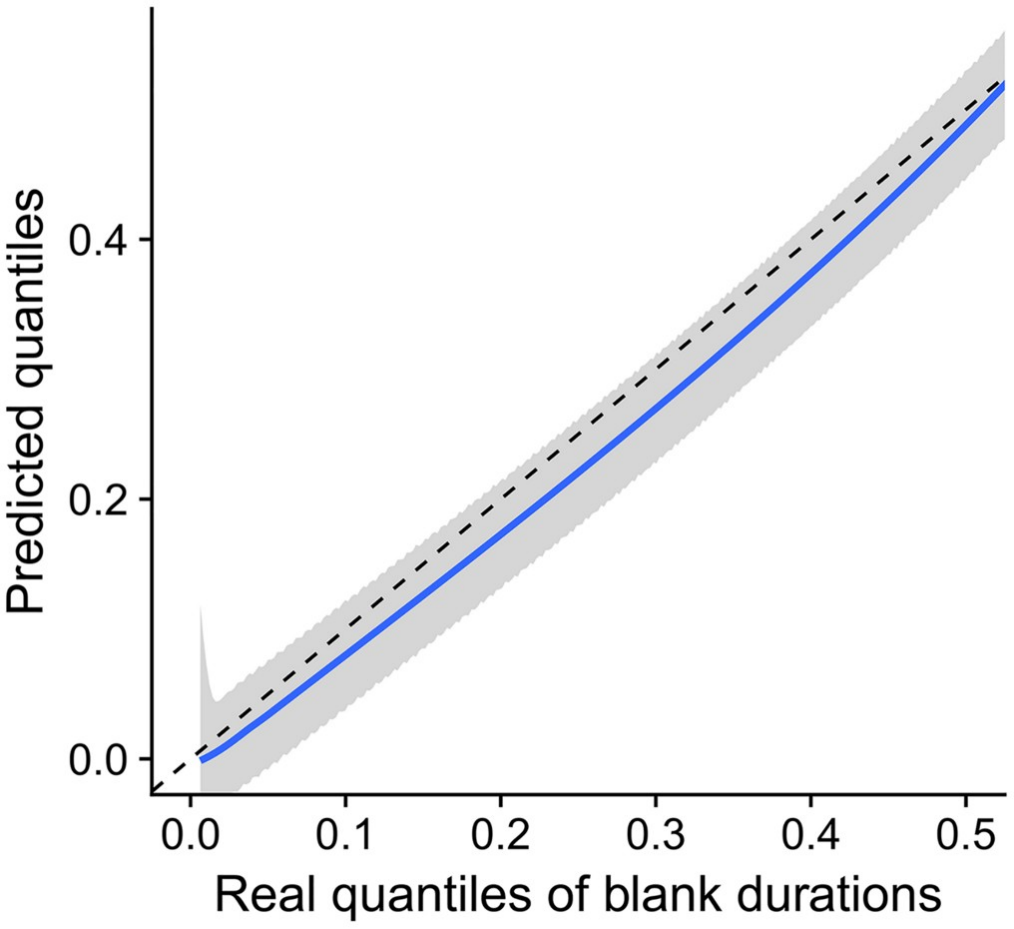
The stimulus-timescale distributions predicted by the information theory for optimal encoding in high S/N scenario (determined from the square root of the Fisher information, the Jeffreys prior), represented by their quantiles *vs*. quantiles of the real blank duration distributions (see also Fig 7). The predicted quantiles (blue line) together with the 95% confidence interval (gray area) are very close to the real quantiles of blanks (dashed line), suggesting near-optimal information transmission.

We constructed stimulus-timescale distributions that would satisfy the definition of the Jeffreys prior, based on the empirical Fisher informations, and compared them with the real stimulus-timescale distributions. In most cases these two appear to be in a close agreement (Figs 7 and 8), which is more evident for short blanks. As the blanks get longer, the predicted distributions decrease more slowly than the real distributions of blanks, however, we should bear in mind that Fisher information is most reliably calculated for short blanks, for which we had most of the data, whereas it may be inaccurate for long blanks due to influential outliers. The real and the predicted stimulus-timescale distribution differ also for observations made at 128 m virtual distance, reasons for which are given in the Discussion.

## Discussion

We demonstrated that responses of ORNs are adjusted to the spatio-temporal statistics of pheromone plumes at variable distances from the source as predicted by the efficient coding hypothesis. This is manifested mainly by the fact that the peak decoding accuracy, quantified by Fisher information, aligns with the most frequent timescale of blanks in the plume. The match of the maximum Fisher information and the mode of the distribution of blanks is less convincing only for the distance of 128 meters, possibly due to two reasons. First, whiffs at 128 m can be relatively very long and ORNs can become temporarily insensitive to the pheromone delivery. Second, neuronal recordings obtained for 128 meters typically contain a smaller number of blanks and whiffs, which can last longer, and therefore fewer responses are available, yielding possibly erroneous estimates of the Fisher information.

We report that not only the peak but also the overall decoding accuracy is adjusted to the local stimulus-timescale statistics. That is, the average decoding accuracy of pheromone encounters with the matching stimulus-timescale statistics of the particular distance is higher than if the same encoding model is applied for the non-matching statistics at a shorter distance. This suggests that there might exist processes, e.g. adaptation, driving ORNs to a response behavior optimal for the local stimulus distribution. Unfortunately, we cannot evaluate the coding accuracy in the non-adjusted state to assess if it improves in time, because the construction of Fisher information requires much more data than can be extracted from the beginnings of ORN recordings. Besides, the dynamic change of neuronal responses at the very beginning of the recordings might also be eventually influenced by the initial dynamics of the pheromone concentration, which can be neither traced nor controlled. Hence, we purposely do not infer the dynamical changes of coding properties, but only the adjusted state.

Another important finding is that the distribution of the stimulus timescale is close to the one that would be a Jeffreys prior with respect to the Fisher information. Such a relationship has important implications from a perspective of information theory [52, 53]. Under the assumption of vanishing response variability, which is essentially the case when many independent noisy “sensors” provide the signal for the decoder, the Jeffreys prior is the optimal stimulus distribution in terms of maximizing the mutual information between stimuli and responses [41–45]. We speculate that such situation in fact corresponds to the anatomy of the moth peripheral olfactory system, where the output of hundreds of ORNs converges onto a single antennal lobe neuron [24, 39]. The optimality of the Jeffreys prior has been theoretically predicted but never actually experimentally observed, to the best of our knowledge.

The fact that the stimulus-timescale distribution is close to the Jeffreys prior might have also some “technical” implications supporting the robustness of the reported results with respect to the chosen unit system of the duration of blanks. It is known that the Fisher information is not invariant with respect to the physical scale on which the stimulus is quantified. It has been demonstrated [54] that the change of the scale may shift the location of the maximum Fisher information, which could disrupt the match with the mode of the stimulus distribution. However, if the stimulus is distributed according to the Jeffreys prior, the match of the two modes is preserved after any arbitrary rescaling, i.e. for any choice of stimulus measurement units. Therefore our observation of the matching peaks of the timescale distributions and the Fisher informations does not depend on the chosen unit system.

Turbulence erases global gradients pointing towards the source, whereas local gradients point in random directions, so that the temporal structure of the sensory input is the unique information about the location of the source. The temporal pattern of odor encounters by a male moth is constantly changing as it flies to the pheromone source. ORNs may constantly adapt their coding to the temporal statistics of the odor signal, a process that would contribute to the efficient tracking by flying moths of pheromone plumes from large distances.

How ORNs dynamically adapt to a particular temporal pattern of odor encounters is elusive as a comprehensive picture of the insect olfactory transduction does not emerge yet. In particular, whether moth pheromone-responding receptors, which belong to the so-called OR family of insect odorant receptors, are ionotropic and/or metabotropic remains a matter of controversial discussion [55–59]. OR-expressing ORNs adapt to strong and/or prolonged stimuli [60]. Adaptation in insect ORNs covers a broad range of timescales, allowing a dynamic adjustment of their responsiveness: *Drosophila* ORNs can adapt to odorant pulses as brief as 35 ms on timescales as fast as 500 ms [61]. In *A. ipsilon*, ORNs exhibit short-term (timescale lower than a second) and long-term adaptation (timescale of minutes) in response to dynamical stimuli [7]. Adaptation occurs both at the level of receptor potential and action potential generators [62, 63]. Sliding adjustment of odor response threshold and kinetics has several molecular actors including ion channels, second messengers and ORs. ORs form non-selective cation channels which are also permeable for Ca^2+^. OR activation leads to Ca^2+^ influx into ORNs. Adaptation in *Drosophila* OR-expressing ORNs is mediated by the Ca^2+^ influx during odor responses [61]. First, Ca^2+^-dependent channels, such as BK channels which underlie the largest current density in moth ORNs [64], may serve for odor adaptation as in vertebrate ORNs [65]. Second, G protein signaling cascades can increase (adenylyl cyclase-dependent signaling [55], phospholipase C-dependent signaling [57]) or decrease (guanylate cyclase-dependent signaling [57]) the ORN sensitivity. Finally, ORs also adjust their sensitivity according to previous odor detections. Insect ORs are heteromers formed by an odor-specific OrX protein and an ubiquitous odorant co-receptor, Orco. Orco plays a central role both in down- and up-regulating the ORN sensitivity. Orco dephosphorylation upon prolonged odor exposure reduces the OR sensitivity [66]. On the other hand, Orco activation that depends on Ca^2+^, Ca^2+^-dependent proteins (protein kinase C and calmodulin) and cAMP production contribute to OR sensitization after moderate odor stimulation [55]. In moth pheromone-sensitive ORNs, Orco was proposed to function as a pacemaker channel, controlling the kinetics of the pheromone responses [67]. In addition, to expand the dynamic range of olfactory detection and thus allow to encode the temporal structure of odor plumes independent of their concentration [68], one or a combination of mechanisms of modulation of ORN sensitivity may contribute to adjust their coding efficiency to temporal statistics of pheromone fluctuations. Ca^2+^ plays a central role in tuning ORN sensitivity and fine adjustments of the Ca^2+^ concentration at the receptor potential and/or spike initiation generator site may be the principal mechanism of this adjustment of coding efficiency.

## Supporting information

S1 Data Recorded spike times of olfactory receptor neurons. (ZIP)
